# F*: an interpretable transformation of the F-measure

**DOI:** 10.1007/s10994-021-05964-1

**Published:** 2021-03-15

**Authors:** David J. Hand, Peter Christen, Nishadi Kirielle

**Affiliations:** 1 Imperial College London, London, UK; 2 School of Computer Science, The Australian National University, Canberra, Australia

**Keywords:** F1-score, Classification, Interpretability, Performance, Error rate, Precision, Recall

## Abstract

The F-measure, also known as the F1-score, is widely used to assess the performance of classification algorithms. However, some researchers find it lacking in intuitive interpretation, questioning the appropriateness of combining two aspects of performance as conceptually distinct as precision and recall, and also questioning whether the harmonic mean is the best way to combine them. To ease this concern, we describe a simple transformation of the F-measure, which we call $F^*$ (F-star), which has an immediate practical interpretation.

## Introduction

Many different measures have been used to evaluate the performance of classification algorithms (see, for example, Chicco and Jurman 2020; Demšar 2006; Ferri et al. 2009; Hand 2012; Powers 2011; Sokolova and Lapalme 2009). Such evaluation is central to choosing between algorithms—to decide which is the best to use in practice, to decide if a method is “good enough”, to optimise parameters (equivalent to choosing between methods), and for other reasons. The data on which such assessments are based is normally a test set that is independent of the training data, consisting of a score and an associated true class label for each object. Here we consider the two-class case, with labels 0 and 1. Objects are assigned to class 1 if their score exceeds some threshold *t*, and to class 0 otherwise. This reduces the data for the evaluation measure to a two-by-two table, the confusion matrix, with counts as shown in Table 1.

**Table 1:** Notation for confusion matrix

		*True class*	
		0	1
*Predicted Class*	0	*TN* (true negatives)	*FN* (false negatives)
	1	*FP* (false positives)	*TP* (true positives)

In general, such a table has four degrees of freedom. Normally, however, the total number of test set cases, $n=TN+FN+FP+TP$, will be known, as will the relative proportions belonging to each of the two classes. These are sometimes called the priors, or the prevalence in medical applications. This reduces the problem to just two degrees of freedom, which must be combined in some way in order to yield a numerical measure on a univariate continuum which can be used to compare classifiers. The choice of the two degrees of freedom and the way of combining them can be made in various ways. In particular, the columns and rows of the table yield proportions which can then be combined (using the known relative class sizes). These proportions go under various names, including, recall or sensitivity, $TP/(TP+FN)$; precision or positive predictive value, $TP/(TP+FP)$; specificity, $TN/(TN+FP)$; and negative predictive value, $TN/(TN+FN)$.

These simple proportions can be combined to yield familiar performance measures, including the misclassification rate, the kappa statistic, the Youden index, the Matthews coefficient, and the F-measure or F1-score (Chicco and Jurman 2020; Hand 2012).

Another class of measures acknowledges that the value of the classification threshold *t* which is to be used in practice may not be known at the time that the algorithm has to be evaluated and when a choice between algorithms has to be made, so that they average over a distribution of possible values of *t*. Such measures include the Area Under the Receiver Operating Characteristic Curve (AUC) (Davis and Goadrich 2006) and the H-measure (Hand 2009; Hand and Anagnostopoulos 2014).

We should remark that the various names are not always used consistently and also that particular measures go under different names, this being a consequence of the widespread applications of the ideas, which arise in many different application domains. An example is the equivalence of recall and sensitivity discussed above.

Many of the performance measures have straightforward intuitive interpretations. For example:

- the misclassification rate is simply the proportion of objects in the test set which are incorrectly classified;
- the kappa statistic is the chance-adjusted proportion correctly classified;
- the H-measure is the fraction by which the classifier reduces the expected minimum misclassification loss, compared with that of a random classifier.

The F-measure is particularly widely used in computational disciplines. It was originally developed in the context of information retrieval to evaluate the ranking of documents retrieved based on a query (Van Rijsbergen 1979). In recent times the F-measure has gained increasing interest in the context of classification, especially to evaluate imbalanced classification problems, in various domains including machine learning, computer vision, data analytics, and natural language processing. It has a simple interpretation as the harmonic mean of the two confusion matrix degrees of freedom: precision, $P=TP/(TP+FP)$, and recall, $R=TP/(TP+FN)$:

1 $$\begin{aligned} F = \frac{2}{\frac{1}{P} + \frac{1}{R}} = \frac{2 P R }{P + R}. \end{aligned}$$

Since precision and recall tap different, and in a sense complementary, aspects of classification performance, it seems reasonable to combine them into a single measure. But averaging them may not be so palatable. One can think of precision as an empirical estimate of the conditional probability of a correct classification given predicted class 1 $(Prob(True=1|Pred=1))$, and recall as being an empirical estimate of the conditional probability of a correct classification given true class 1 $(Prob(Pred=1|True=1))$. An average of these has no interpretation as a probability.

Moreover, despite the seminal work of Van Rijsbergen (1979), some researchers are uneasy about the use of the harmonic mean (Hand and Christen 2018), preferring other forms of average (e.g. an arithmetic or geometric mean). For example, the harmonic mean of two values has the property that it lies closer to the smaller of the values than the larger. In particular, if one of recall or precision is zero, then the harmonic mean (and therefore *F*) is zero, ignoring the value of the other. The desire for an interpretable perspective on *F* has been discussed, for example, on Stack Exchange (2013).

In an attempt to tackle this unease, in what follows we present a transformed version of the F-measure which has a straightforward intuitive interpretation.

## The F-measure and F*

Plugging the counts from Table 1 into the definition of *F*, we obtain

$$\begin{aligned} F = \frac{2}{\frac{TP+FP}{TP} + \frac{TP+FN}{TP}} = \frac{2 TP}{FN+FP+2TP}, \end{aligned}$$

from which

$$\begin{aligned} \frac{TP}{FN+FP} = \frac{1}{2} \frac{F}{1-F}. \end{aligned}$$

So if we define $F^{\prime}$ as $F^{\prime} = F / 2(1-F)$, we have that $F^{\prime}$ is *the number of class 1 objects correctly classified for each object that is misclassified*.

This is a straightforward and attractive interpretation of a transformation of the F-measure, and some researchers might prefer to use it. However, $F^{\prime}$ has the property that it is a ratio and not simply a proportion, so it is not constrained to lie between 0 and 1—as are most other performance measures.

We can overcome this by a further transformation, yielding

2 $$\begin{aligned} \frac{TP}{FN+FP+TP} = \frac{F}{2-F}. \end{aligned}$$

Now, defining $F^*$ (F-star) as $F^* = F/(2-F),$^1^ we have that:

${{F}}^{*}$ *is the proportion of the relevant classifications which are correct, where a relevant classification is one which is either really class 1 or classified as class 1.*

Under some circumstances, researchers might find alternative ways of looking at $F^*$ useful. In particular:

- $F^*$ *is the number of correctly classified class 1 objects expressed as a fraction of the number of objects which are either misclassified or are correctly classified class 1 objects*; or,
- $F^*$ *is the number of correctly classified class 1 objects expressed as a proportion of the number of objects which are either class 1, classified as class 1, or both*; or, yet a fourth alternative,
- $F^*$ *is the number of correctly classified class 1 objects expressed as a fraction of the number of objects which are not correctly classified class 0 objects*.

$F^*$ can be alternatively written as $F^*=TP/(n-TN)$, which can be directly calculated from the confusion matrix. Researchers may recognise this as the Jaccard coefficient, widely used in areas where true negatives may not be relevant, such as numerical taxonomy and fraud analytics (Jaccard 1908; Dunn and Everitt 1982; Baesens et al. 2015).

**Fig. 1:**
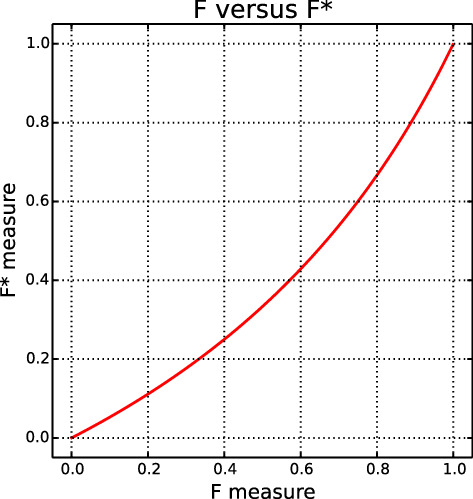
The transformation from *F* to $F^*$

To illustrate, if class 1 objects are documents in information retrieval, then $F^*$ is the number of relevant documents retrieved expressed as a proportion of all documents except non-retrieved irrelevant documents. Or, if class 1 objects are COVID-19 infections, then $F^*$ is the number of infected people who test positive divided by the number who either test positive or are infected or both.

**Fig. 2:**
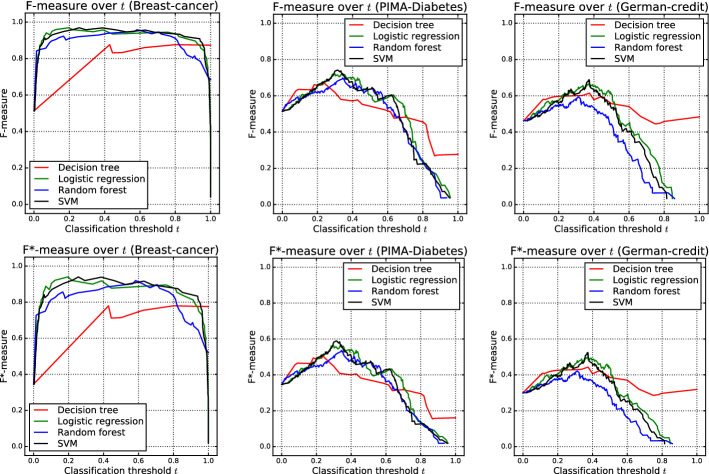
Experimental results showing the *F* (top) and $F^*$ (bottom) measure on three public data sets using four classification techniques

The relationship between $F^*$ and *F* is shown in Fig. 1. The approximate linearity of this curve shows that $F^*$ values will be close to *F* values. More importantly, however, is the fact that $F^*$ is a monotonic transformation of *F*. This means that any conclusions reached by seeing which $F^*$ values are larger will be identical to those reached by seeing which *F* values are larger. In particular, choices between algorithms will be the same. This is illustrated in Fig. 2, which shows experimental results for three public data sets from the UCI Machine Learning Repository (Lichman 2013) for four classifiers as implemented using Sklearn (Pedregosa et al. 2011) with default parameter settings and the classification threshold *t* varying between 0 and 1. Although the curve shapes differ slightly between $F^*$ and *F* (because of the monotonic *F* to $F^*$ transformation of the vertical axis), the threshold values at which they cross are the same.

Van Rijsbergen (1979) also defines a weighted version of *F*, placing different degrees of importance on precision and recall. This carries over immediately to yield weighted versions of both $F^{\prime}$ and $F^*$.

## Discussion

The overriding concern when choosing a measure of performance in supervised classification problems should be to match the measure to the objective. Different measures have different properties, emphasising different aspects of classification algorithm performance. A poor choice of measure can lead to the adoption of an inappropriate classification algorithm, in turn leading to suboptimal decisions and actions.

A distinguishing characteristic of the F-measure is that it makes no use of the *TN* count in the confusion matrix—the number of class 0 objects correctly classified as class 0. This can be appropriate in certain domains, such as information retrieval where *TN* corresponds to irrelevant documents that are not retrieved, fraud detection where the number of unflagged legitimate transactions might be huge, data linkage where there generally is a large number of correctly unmatched record pairs that are not of interest, and numerical taxonomy where there is an unlimited number of characteristics which do not match for any pair of objects. In other contexts, however, such as in medical diagnosis, correct classification to each of the classes can be important.

The F-measure uses the harmonic mean to combine precision and recall, two distinct aspects of classification algorithm performance, and some researchers question the use of this form of mean and the interpretability of their combination. In this paper, we have shown that suitable transformations of *F* have straightforward and familiar intuitive interpretations. Other work exploring the combination of precision and recall includes Goutte and Gaussier (2005), Powers (2011), Boyd et al. (2013) and Flach and Kull (2015).

## Footnotes

1 In terms of precision and recall, $F^* = P R / (P+R-P R)$.
